# Nuclear Retention of Multiply Spliced HIV-1 RNA in Resting CD4^+^ T Cells

**DOI:** 10.1371/journal.ppat.0020068

**Published:** 2006-07-07

**Authors:** Kara G Lassen, Kasra X Ramyar, Justin R Bailey, Yan Zhou, Robert F Siliciano

**Affiliations:** 1 Department of Medicine, Johns Hopkins University School of Medicine, Baltimore, Maryland, United States of America; 2 Howard Hughes Medical Institute, Baltimore, Maryland, United States of America; King's College London, United Kingdom

## Abstract

HIV-1 latency in resting CD4^+^ T cells represents a major barrier to virus eradication in patients on highly active antiretroviral therapy (HAART). We describe here a novel post-transcriptional block in HIV-1 gene expression in resting CD4^+^ T cells from patients on HAART. This block involves the aberrant localization of multiply spliced (MS) HIV-1 RNAs encoding the critical positive regulators Tat and Rev. Although these RNAs had no previously described export defect, we show that they exhibit strict nuclear localization in resting CD4^+^ T cells from patients on HAART. Overexpression of the transcriptional activator Tat from non-HIV vectors allowed virus production in these cells. Thus, the nuclear retention of MS HIV-1 RNA interrupts a positive feedback loop and contributes to the non-productive nature of infection of resting CD4^+^ T cells. To define the mechanism of nuclear retention, proteomic analysis was used to identify proteins that bind MS HIV-1 RNA. Polypyrimidine tract binding protein (PTB) was identified as an HIV-1 RNA-binding protein differentially expressed in resting and activated CD4^+^ T cells. Overexpression of PTB in resting CD4^+^ T cells from patients on HAART allowed cytoplasmic accumulation of HIV-1 RNAs. PTB overexpression also induced virus production by resting CD4^+^ T cells. Virus culture experiments showed that overexpression of PTB in resting CD4^+^ T cells from patients on HAART allowed release of replication-competent virus, while preserving a resting cellular phenotype. Whether through effects on RNA export or another mechanism, the ability of PTB to reverse latency without inducing cellular activation is a result with therapeutic implications.

## Synopsis

HIV-1 has the ability to establish a state of latent infection in resting memory CD4^+^ T cells. These latently infected cells represent a stable reservoir for the virus that is a major barrier to viral eradication. Understanding how this reservoir is established, maintained, and reactivated is essential for developing methods to target and eliminate these cells. Currently, many proposed mechanisms of HIV-1 latency involve a dramatic reduction in ongoing HIV-1 transcription. However, some HIV-1 mRNAs are made, and it has been unclear why the cells are unable to produce virus. This study describes the surprising observation that mRNAs encoding the viral regulatory proteins Tat and Rev are retained in the nucleus of infected resting CD4^+^ T cells. A cellular HIV-1 RNA-binding protein called polypyrimidine tract binding protein was shown to reverse latency when overexpressed in resting CD4^+^ T cells. This overexpression of polypyrimidine tract binding protein was sufficient to allow release of replication-competent HIV-1 from latently infected cells without inducing cellular stimulation. These experiments suggest that multiple factors contribute to the maintenance of HIV-1 latency in vivo; however, perturbation of the level of a specific cellular protein is sufficient to overcome these blocks and allow for virus production.

## Introduction

Treatment of HIV-1-infected individuals with highly active antiretroviral therapy (HAART) can reduce plasma virus levels to below the limit of detection of ultra-sensitive clinical assays [1–3]. However, even in the setting of optimal treatment, replication-competent HIV-1 persists in resting CD4^+^ T cells [4–8] and possibly in other viral reservoirs (reviewed in [9]). Resting CD4^+^ T cells from patients on HAART do not spontaneously produce HIV-1 unless activated [4,10]. However, following activation of these cells, replication-competent HIV-1 can be invariably recovered even from patients who have had suppression of viremia on HAART for as long as seven years [11,12]. Taken together, these results demonstrate that a stable state of latent infection can be established in resting CD4^+^ T cells. The latent reservoir has an extremely slow decay rate [11–15] that will likely preclude virus eradication unless novel approaches [16–23] can purge latently infected cells. Of particular interest are strategies that would induce latent HIV-1 without causing global T-cell activation [20]. The design of such strategies requires an understanding of the molecular mechanisms of latency.

Resting CD4^+^ T cells from infected individuals contain rare cells with integrated HIV-1 DNA [4,5,24], and these cells are presumed to represent the stable latent reservoir, since most studies indicate that unintegrated forms of HIV-1 DNA are labile [25–28]. Among cells with integrated HIV-1 DNA, only a small fraction can be induced to release replication-competent virus following cellular activation [5]. The rest contain defective or permanently silenced viral genomes. Mechanistic studies of latency are thus complicated by the fact that latently infected cells (cells capable of releasing replication-competent virus) represent only a small fraction of the cells carrying HIV-1 DNA, which in turn represent only a small fraction of the resting CD4^+^ T cell population. Mechanistic studies of HIV-1 latency must be interpreted with these caveats in mind. Because of the difficulties involved in the analysis of HIV-1 latency in vivo, many mechanistic studies have been carried out in cell line systems that may not precisely reflect the physiology of the profoundly quiescent cells that harbor latent HIV-1 in vivo.

Most of the proposed mechanisms for HIV-1 latency operate at the level of transcription. These include proviral integration into sites that are repressive for transcription [29–32], DNA and histone modifications that inhibit transcription [33,34], the absence of host transcriptional activators necessary for HIV-1 gene expression [35–39], the presence of transcriptional repressors [34,40], transcriptional interference [32,41,42], and the premature termination of HIV-1 transcripts due to the absence of the viral protein Tat and Tat-associated host factors that are critical for efficient transcriptional elongation [43–47]. There are clearly major defects in transcriptional initiation and elongation in resting CD4^+^ T cells [21,44,48,49], and processive HIV-1 transcripts are difficult to detect in these cells [50,51].

In addition to these mechanisms affecting transcription, post-transcriptional mechanisms may also contribute to latency. With extremely sensitive methods, some processive transcripts, including both unspliced (US) and multiply spliced (MS) HIV-1 mRNAs, have been demonstrated in resting CD4^+^ T cells from infected individuals [10,49]. Despite the presence of these transcripts, no virus is produced. This could reflect deficient nuclear export of incompletely spliced HIV-1 RNAs in the absence of the viral protein Rev [52,53]. Interestingly, Rev and the transcriptional activator Tat, are both products of MS HIV-1 RNAs, which have no known export defect. An absence of each of these viral proteins has been implicated in latency [21,47,52,53]. Tat acts at the level of transcription elongation to promote high level transcription from the HIV-1 long terminal repeat (LTR) [43,46,54,55], while Rev promotes export of incompletely spliced viral HIV-1 RNAs to the cytoplasm [56–59]. In cells from patients on HAART, exogenous Tat has been shown to promote positive feedback upregulation of HIV-1 gene expression in cells from infected individuals [21], and a recent study in infected cell lines has shown that stochastic fluctuations in Tat activity can lead to large differences in the levels of HIV-1 gene expression [47]. Given the presence of MS HIV-1 mRNAs encoding Tat and Rev in resting CD4^+^ T cells [49], it has been unclear why positive feedback does not increase virus gene expression to levels sufficient for virus production. We describe here a post-transcriptional block in the export of MS HIV-1 RNA that may prevent this positive feedback loop. In addition, we identify a host protein which, when overexpressed in resting CD4^+^ T cells, can reverse latency without inducing cellular activation.

## Results

### Nuclear Localization of MS HIV-1 RNA in Resting CD4^+^ T Cells

In vivo, latent viral genomes are found in G_0_ resting memory CD4^+^ T cells [5,60]. Because latency may involve aspects of the unique physiology of these profoundly quiescent cells, it is best analyzed using primary resting CD4^+^ T cells from infected individuals rather than transformed cell lines. However, the analysis of latency in primary cells is complicated by the fact that only a small fraction of the cells harboring HIV-1 DNA in vivo can be induced to produce infectious virus upon cellular activation [5]. The remaining cells may carry defective viruses. In the molecular analysis of HIV-1 DNA or RNA species in primary cell populations, it is not generally possible to distinguish replication-competent from defective forms. Replication-competence can only be established in separate virus outgrowth experiments. By combining data from these two approaches, inferences can be made about the biology of the small subpopulation of cells that harbor replication-competent virus.

**Figure 1:**
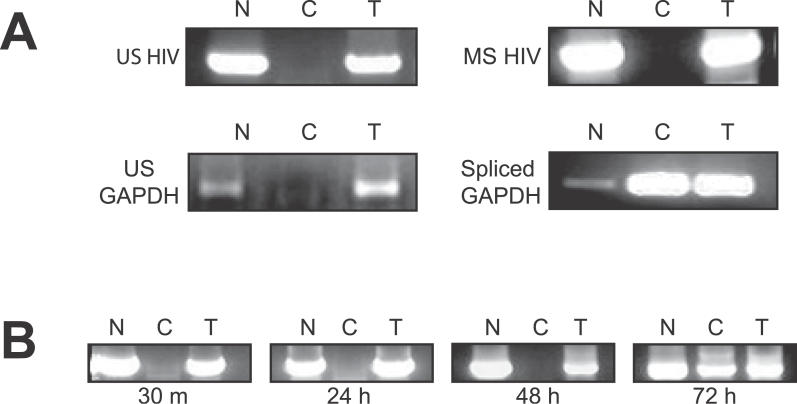
Both MS and US HIV-1 RNAs Localize to the Nucleus of Resting CD4^+^ T Cells from Patients on HAART (A) RT-PCR analysis of US and MS HIV-1 RNAs in nuclear (N), cytoplasmic (C), and total (T) RNA fractions isolated from 10^6^ purified resting CD4^+^ T cells from a patient on HAART. Unspliced GAPDH and spliced GAPDH were used as internal controls for fraction purity and RNA integrity. (B) Effect of cellular activation on the localization of HIV-1 RNAs. 10^6^ resting CD4^+^ T cells were stimulated with anti-CD3 and anti-CD28. Nuclear (N), cytoplasmic (C), and total (T) RNA fractions were isolated at given times post-activation and analyzed as above with primers to detect US (shown) and MS (not shown) HIV-1 RNAs. Similar results were obtained with both US and MS primer sets.

We first analyzed the distribution of the bulk population of HIV-1 RNA molecules in highly purified populations of resting CD4^+^ T cells from patients on suppressive HAART regimens. Both MS and US HIV-1 mRNAs can be detected in these cells with sensitive methods [49]. To understand why the presence of MS HIV-1 mRNA does not lead to Tat-mediated positive feedback amplification of virus gene expression and virus production, we first examined the subcellular localization of HIV-1 RNA in resting CD4^+^ T cells. Nuclear, cytoplasmic, and total RNA fractions were isolated from resting CD4^+^ T cells, and an ultra-sensitive hemi-nested RT-PCR was performed on each fraction. US HIV-1 mRNAs, which have an export defect in the absence of high levels of the HIV-1 Rev protein [52,53], were retained in the nucleus of these cells (Figure 1A). Surprisingly, MS HIV-1 mRNAs, not previously known to have an export defect, were also exclusively nuclear in localization (Figure 1A). The same result was observed in ten patients; both US and MS HIV-1 RNAs were detected only in the nuclear fraction of primary resting CD4^+^ T cells.

Unspliced and spliced glyceraldehhyde-3-phosphate dehydrogenase (GAPDH) mRNAs were also amplified from these fractions in order to determine the quality of the nuclear-cytoplasmic separation, as well as the integrity of the isolated RNA (Figure 1A). As expected, unspliced GAPDH RNA was localized to the nucleus while the majority of spliced GAPDH mRNA was localized to the cytoplasm of resting CD4^+^ T cells. This confirms the efficacy of the cell fractionation, and further demonstrates that the aberrant localization of HIV-1 RNA is not due to a global defect in RNA export in these cells. Whether the RNA localization patterns observed in bulk populations of resting cells from infected donors are characteristic of the tiny fraction of cells harboring latent, replication-competent HIV-1 genomes cannot be determined from this analysis.

To determine whether nuclear localization of HIV-1 RNA is altered in response to T-cell stimulation, purified resting CD4^+^ T cells were activated with anti-CD3/anti-CD28 for various times and then fractionated. Both MS and US HIV-1 RNAs were localized exclusively to the nucleus until 48 to 72 h following stimulation when cytoplasmic HIV-1 RNAs were detected (Figure 1B). The appearance of HIV-1 RNA in the cytoplasm preceded the release of virus particles into the supernatant by 24–48 h [49].

### Identification of MS HIV-1 RNA-Binding Proteins in Resting and Activated CD4^+^ T Cells

The aberrant nuclear localization of MS HIV-1 mRNAs in resting CD4^+^ T cells could be due to the presence of a negative regulator or the absence of a positive export factor. Primary resting and activated CD4^+^ T cells were therefore screened for factors that bind specifically to a minimal MS HIV-1 RNA (Figure S1A). Candidate HIV-1 RNA-binding proteins were then identified by PAGE-mass spectrometry (Figure S1B). This screen identified a number of known RNA-binding proteins, including several which have been previously shown to bind HIV-1 RNA. One of these was polypyrimidine tract binding protein (PTB). PTB plays a role in post-transcriptional regulation of gene expression (reviewed in [61]). For example, PTB is involved in post-transcriptional regulation of gene expression in response to T-cell stimulation [62–64]. PTB has been previously shown to associate with HIV-1 RNA [65]. The role of PTB in HIV-1 gene expression has not yet been defined.

**Figure 2:**
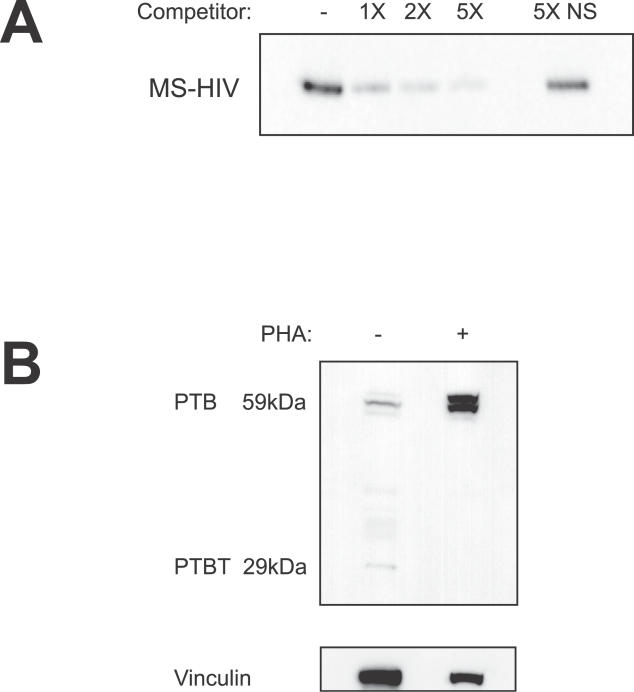
PTB Binds Specifically to MS HIV-1 RNA and Is Upregulated upon Cellular Stimulation (A) PTB binds specifically to MS HIV-1 RNA. Recombinant PTB was pre-incubated with increasing molar equivalents (0X, 1X, 2X, 5X) of non-biotinylated MS HIV-1 RNA (relative to biotinylated MS HIV-1 RNA) or a 5-fold mass excess of non-specific (NS) competitor, total yeast RNA; and then incubated with biotinylated, in vitro-transcribed MS HIV-1 RNA bound to streptavidin-conjugated magnetic particles. PTB protein eluted by RNase digestion was analyzed by Western blot. (B) PTB expression increases in response to T-cell stimulation. Expression of PTB was measured by Western blotting in resting CD4^+^ T cells cultured for 72 h without activating stimuli (−) or with (+) anti-CD3/anti-CD28. Vinculin served as a loading control.

To examine the specificity of PTB binding to HIV-1 RNA, recombinant PTB was incubated with in vitro-transcribed, biotinylated MS HIV-1 RNA bound to streptavidin-conjugated magnetic beads (Figure S1A). Recombinant PTB bound MS HIV-1 RNA and was released upon RNase digestion (Figure 2A). The binding was blocked by pre-incubation of PTB with increasing amounts of non-biotinylated MS HIV-1 RNA. Binding of PTB decreased progressively with increasing ratios of non-biotinylated to biotinylated MS HIV-1 RNA. PTB binding was not decreased by a 5-fold mass excess of total yeast RNA, a non-specific competitor (Figure 2A).

To determine whether differences in PTB levels in resting and activated CD4^+^ T cells might be involved in the observed export defect, we first measured steady-state levels of PTB protein in purified primary resting CD4^+^ T cells and phytohemagglutinin (PHA)-stimulated, activated CD4^+^ T cells. Quantitative Western analyses indicated that PTB expression increases by at least 4.5-fold upon cell stimulation (Figure 2B). PTB has four known isoforms generated by alternative splicing. Three variants produce bands of 57–59 kDa, while the fourth variant of PTB has an apparent molecular weight of 25 kDa. Only the larger isoforms were upregulated following cellular stimulation, while total levels of the 25-kDa isoform (PTBT) decreased upon cellular stimulation (Figure 2B).

### PTB Overexpression Is Sufficient to Cause Virus Production by Resting CD4^+^ T Cells from Infected Individuals

**Figure 3:**
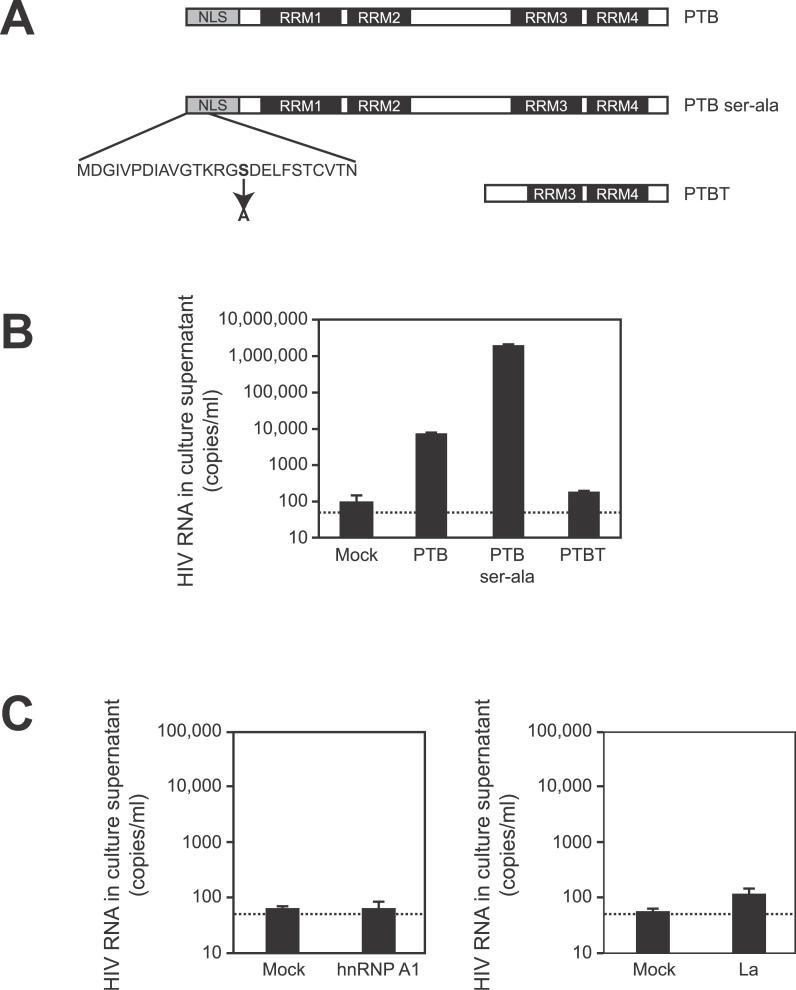
PTB Overexpression Is Sufficient to Reverse HIV-1 Latency in Primary Resting CD4^+^ T Cells (A) Diagram of expression constructs. The PTB construct contains full-length PTB including a NLS and four RNA recognition motifs (RRM). PTB ser-ala has a single serine to alanine substitution at position 16 in the NES located within the NLS region. PTBT is the 25 kDA isoform which lacks an NLS but contains two intact RRMs. (B) Overexpression of PTB and PTB ser-ala is sufficient to upregulate virus production from resting CD4^+^ T cells. Purified resting CD4^+^ T cells from patients on HAART were transfected with the indicated constructs. Culture supernatants were isolated 72 h post-transfection and analyzed for viral RNA. Dotted line represents the limit of detection of the assay. (C) Other cellular proteins that bind HIV-1 RNA cannot induce virus production from resting CD4^+^ T cells of patients on HAART. Resting CD4^+^ T cells were transfected as described above with empty vector or hnRNP A1 (left panel) and empty vector or La (right panel). HIV-1 RNA levels in the supernatants 72 h after transfection are shown.

The direct binding of PTB to MS HIV-1 RNA, and the differential regulation of PTB isoforms with T-cell stimulation, suggested that PTB might act as a positive factor for HIV-1 gene expression. To test this hypothesis, we generated expression constructs for various forms of PTB (Figure 3A). These included full-length PTB and a mutant form of full-length PTB with a serine to alanine mutation in the nuclear export signal (PTB ser-ala). The N-terminal 55 residues of PTB contain a nuclear localization signal (NLS). PTB ser-ala has a single serine to alanine substitution at position 16 in the nuclear export signal that is also located in this region. This mutation dramatically reduces nucleo-cytoplasmic shuttling of PTB but does not alter nuclear import [66]. We also generated an expression vector for PTBT, the 25-kDA isoform, which lacks an NLS but contains two intact RNA recognition motifs that allow it to interact with cognate RNAs as efficiently as full-length PTB does [64].

Resting CD4^+^ T cells from patients on HAART were transfected with empty vector, PTB, PTB ser-ala, or PTBT using an Amaxa Nucleofector. At 72 h post-transfection, an ultra-sensitive RT-PCR assay was employed to detect the low levels of virions released into the supernatants of these cultures. In cells transfected with empty vector or PTBT, the amount of virus released was often below the limit of detection (<50 copies of HIV-1 RNA/ml), although occasionally a mock-treated sample would yield a very low-level positive value for HIV-1 RNA (Figure 3B). Because an undetectable viral RNA measurement could represent low levels of virus production and not an actual zero, we considered an undetectable viral load as equivalent to 50 copies/ml when calculating release. The low positive values may represent non-specific stimulation due to in vitro culture. Cells transfected with PTB or PTB ser-ala showed a dramatic (100- to 10,000-fold) upregulation of virus production (Figure 3B). The stimulation of HIV-1 production by PTB transfection was consistently observed in all five patients tested. Although these results do not establish that the virions released are infectious, they do show that PTB overexpression can induce infected resting CD4^+^ T cells to produce and release virus particles. To determine if the observed upregulation of virus production was specific to PTB, other HIV-1 RNA-binding proteins identified in the proteomic screen were overexpressed in resting CD4^+^ T cells. Cells transfected with hnRNPA1 or La, other host proteins capable of binding HIV-1 RNA [65,67,68], did not upregulate viral gene expression (Figure 3C).

### PTB Expression Specifically Upregulates HIV-1 Gene Expression without Inducing Cellular Activation

**Figure 4:**
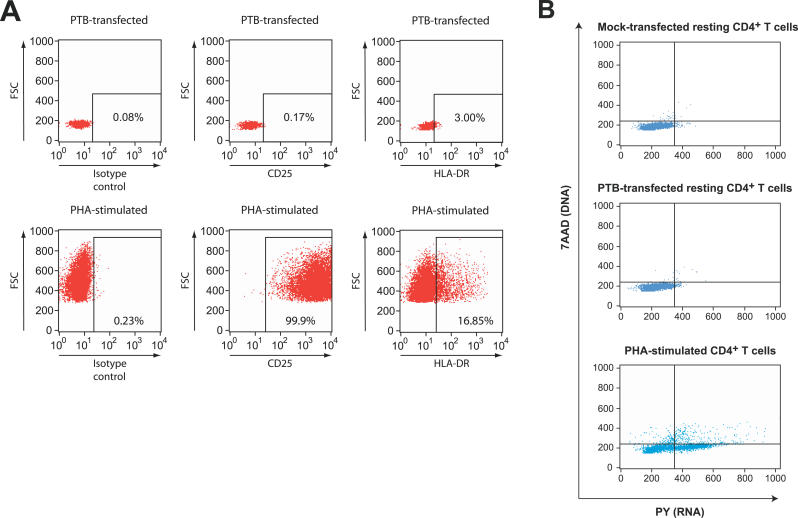
PTB Expression Specifically Upregulates HIV-1 Gene Expression without Inducing Cellular Activation (A) Expression of activation markers on PTB-transfected primary resting CD4^+^ T cells. PTB-transfected cells were analyzed 24, 48 (shown), and 72 h post-transfection for surface expression of CD25 and HLA-DR. Cells were stained with phycoerythrin-conjugated isotype control antibody, anti-CD25, or anti-HLA-DR. Cell size is indicated by the forward scatter (FSC) values on the Y-axis. (B) PTB-transfected cells do not transition to the G_1b_ stage of the cell cycle. Mock-transfected and PTB-transfected cells and PHA-stimulated cells were stained with PY and 7AAD to measure RNA and DNA levels, respectively. Dye binding was assessed by flow cytometry.

To determine if PTB overexpression was inducing virus production non-specifically through general effects on the activation status of the transfected cells, we carefully evaluated transfected cells for expression of well-established markers of cellular activation. PTB-transfected resting CD4^+^ T cells did not express the activation marker CD25, and only a small fraction (3%) of the cells expressed levels of HLA-DR that were above background (Figure 4A). Following PTB transfection, there was no detectable upregulation of CD69, an early activation marker, at 8 h post-transfection (not shown). In addition, PTB-transfected cells maintained a small, resting cell morphology as shown by the forward scatter profiles in Figure 4A and by the photomicroscopy (see below). As a positive control, we showed that expression of CD25 and HLA-DR were markedly increased by PHA stimulation, as was cell size (Figure 4A). Additionally, transfected cells remained in the G_0_/G_1a_ phase of the cell cycle. Transfected cells were stained with 7-amino-actinomycin-D/pyronin Y (7AAD/PY) to evaluate DNA/RNA content and cell-cycle status. As is shown in Figure 4B, PTB transfection caused no change in DNA levels and only a slight increase in RNA levels. In contrast, PHA activation caused a dramatic increase in cellular RNA levels followed by entry of cells into the cell cycle. Taken together, these results suggest that overexpression of PTB in resting CD4^+^ T cells can induce virus production without the wholesale induction of the G_0_/G_1a_ → G_1b_ transition that is associated with increased permissiveness to HIV-1 replication [69,70].

### High Level Expression of Tat or PTB Results in Efficient Virus Production Comparable to Virus Production by PHA-Stimulated Resting CD4^+^ T Cells

**Figure 5:**
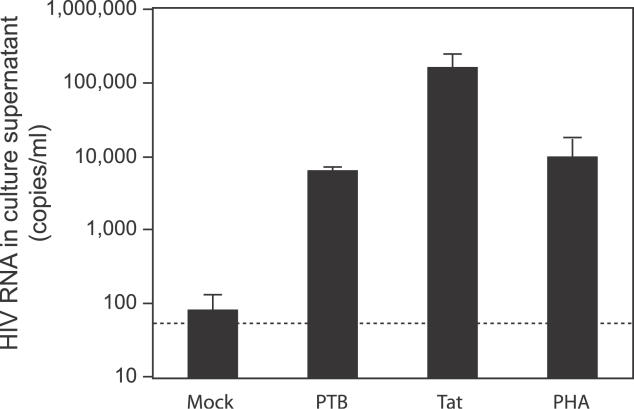
Induction of Virus Production by PTB or Tat High-level expression of PTB or Tat in resting CD4^+^ T cells from patients on HAART induces virus production that is comparable to that seen following PHA stimulation. Resting CD4^+^ T cells purified from patients on HAART were transfected as described above with expression vectors for PTB or Tat, or as a positive control, were stimulated with PHA. For all transfections, supernatant HIV-1 levels were measured at 72 h post-stimulation. For PHA-stimulated cells, supernatant levels of HIV-1 RNA were measured every day for a 6-d period. Levels shown reflect peak virus release over the 6-d period.

The results presented above suggest that the export block affecting MS HIV-1 RNAs encoding the Tat and Rev may contribute to the non-productive nature of HIV-1 infection of resting CD4^+^ T cells by blocking a positive feedback loop that would result if these RNAs could be translated in the cytosol. To further test this hypothesis, we bypassed this block by transfecting resting CD4^+^ T cells from patients on HAART with a simple, non-HIV expression vector carrying a Tat cDNA. Tat expressed in this context caused dramatic (1,000-fold) upregulation of virus production (Figure 5) that was even greater than that seen in PTB-transfected cells or in cells stimulated with the mitogen PHA. This result is consistent with previous observations that infected resting cells have a defect in transcriptional elongation [21,44,48,49], and that this defect can be overcome by Tat [21]. These findings can now be reconciled with the observation that resting cells express MS mRNA for Tat and Rev without producing virus [49]. Although MS RNAs encoding these positive regulatory factors are produced at low levels in resting CD4^+^ T cells, they are retained in the nucleus, and thus no positive feedback is possible. This effect is reversed by increased levels of PTB or a dramatic increase in transcription from the HIV-1 LTR.

### Partial Knockdown of PTB in Activated CD4^+ ^T Cells Is Not Sufficient to Prevent Virus Production

Because overexpression of Tat alone in resting CD4^+^ T cells is sufficient to upregulate viral gene expression in infected resting CD4^+^ T cells (Figure 5), we suspected that the export defect in resting CD4^+^ T cells may be saturated and overcome at high levels of HIV-1 gene expression. Thus, partial short interfering RNA (siRNA) knockdown of PTB in maximally activated CD4^+^ T cells did not decrease HIV-1 production (Figure S2). PTB may be overcoming a negative factor affecting HIV-1 RNA export that is readily saturable or that is present only in resting CD4^+^ T cells.

### Nuclear Expression of PTB Is Required for Efficient HIV-1 Gene Expression

**Figure 6:**
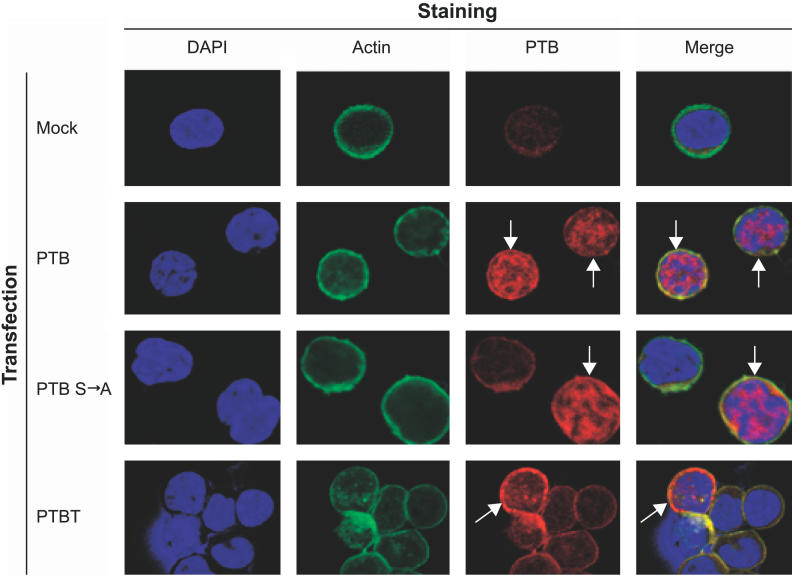
PTB Localization in Transfected Primary Cells Resting CD4^+^ T cells were mock-transfected or were transfected with the expression construct indicated on far left and stained as indicated on the top. Cells were stained with DAPI (blue) and with antibodies to actin (green) and PTB (red). Immunofluorescence of transfected resting CD4^+^ T cells demonstrated strong nuclear localization of both PTB and PTB ser-ala (speckled pink/blue pattern in nucleus). Transfected cells are indicated with arrows.

Because MS HIV-1 mRNAs have a nuclear export defect in resting CD4^+^ T cells (Figure 1), we hypothesized that only nuclear forms of PTB could interact with MS HIV-1 mRNA and facilitate export. Therefore, the nuclear localization of PTB isoforms was examined in primary resting CD4^+^ T cells by immunofluorescence using an antibody that recognizes all isoforms. In untransfrected cells, endogenous PTB staining was largely cytoplasmic (Figure 6, top panels). This result likely reflects the prominence in resting cells of the PTBT isoform that lacks an NLS and that shows cytoplasmic localization in other cells [64]. Similarly, in PTBT-transfected cells, the majority of PTBT was localized to the cytoplasm (Figure 6, bottom panels). In contrast, cells transfected with PTB exhibited strong nuclear localization (Figure 6, center panels). PTB ser-ala, which carries a disrupted nuclear export signal, also showed strong nuclear localization. Interestingly, cells transfected with PTB ser-ala consistently showed the highest levels of virus production (Figure 3). Taken together, these results suggest that nuclear localization of PTB is important for upregulation of virus production by infected resting CD4^+^ T cells.

### PTB Overexpression Does Not Increase Total Intracellular Concentrations of HIV-1 RNA

**Figure 7:**
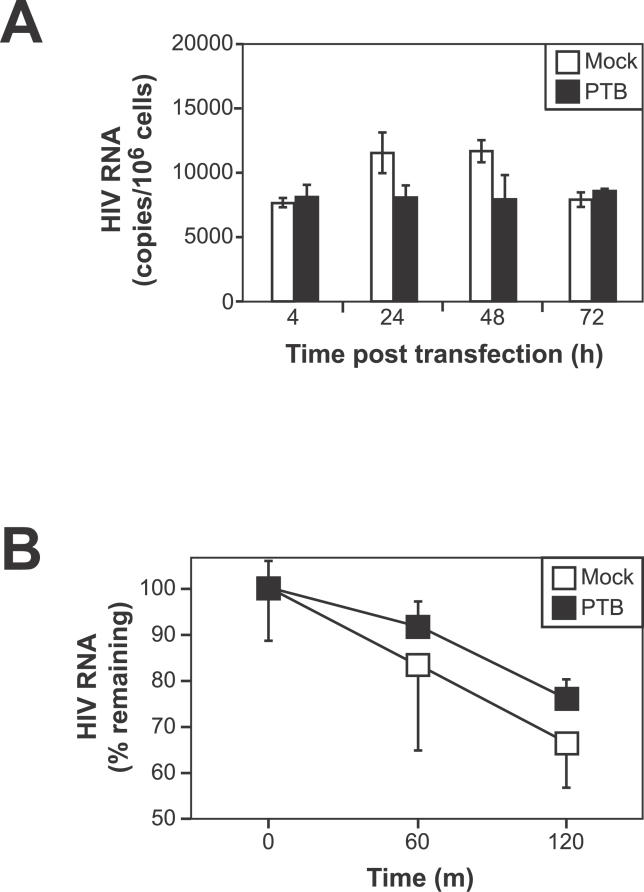
Overexpression of PTB Does Not Increase in Total Intracellular Concentrations of HIV-1 RNA (A) Steady-state levels of HIV-1 RNA are not increased by overexpression of PTB. Resting CD4^+^ T cells (4 × 10^6^) were transfected as described above. 1 × 10^6^ cells were isolated at various times post-transfection. Total cellular RNA was isolated, and quantitative real-time RT-PCR was performed using primers that detect total HIV-1 transcripts. (B) PTB has no major effect on HIV-1 RNA stability. Resting CD4^+^ T cells (4 × 10^6^) were transfected as described above. At 24 h post-transfection, α-amanitin was added to cells to block de novo RNA synthesis. Total RNA was isolated at given times and real-time RT-PCR was performed to detect total HIV-1 RNAs.

We next asked how nuclear forms of PTB stimulate virus production. In other systems, PTB acts post-transcriptionally through effects on RNA processing and stability [61,62,64]. We measured total steady-state levels of intracellular HIV-1 RNA following transfection of resting CD4^+^ T cells from patients on HAART with PTB or empty vector. PTB transfection had no observed effect on total intracellular HIV-1 RNA levels compared to mock-transfected controls (Figure 7A). PTB has also been shown previously to stabilize certain cellular RNAs [64]. To examine the possibility that PTB alters the half-life of intracellular HIV-1 RNAs, we measured the turnover of HIV-1 RNAs in cells transfected with PTB (Figure 7B). At 24 h post-transfection, α-amanitin was added to cells to block de novo RNA synthesis. Total RNA was isolated at given times, and real-time RT-PCR was performed to detect total HIV-1 RNAs. Transfection with PTB did not substantially alter the turnover rate of HIV-1 RNAs compared with mock-transfected controls.

### PTB Expression Alters the Subcellular Distribution of HIV-1 RNAs in Resting CD4^+^ T Cells In Vivo

**Figure 8:**
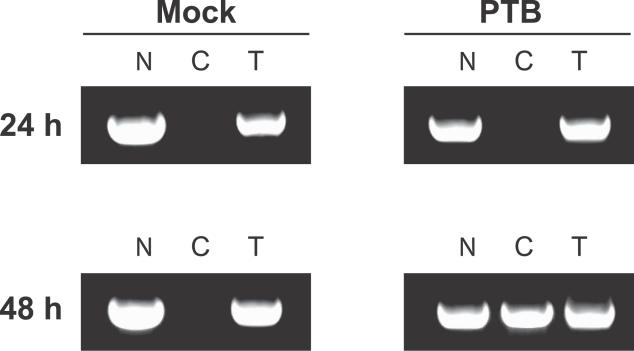
PTB Expression Alters the Subcellular Distribution of HIV-1 RNAs in Primary Resting CD4^+^ T Cells from patients on HAART Nuclear (N), cytoplasmic (C), and total (T) RNA fractions were isolated from mock-transfected (left) or PTB-transfected (right) resting CD4^+^ T cells 24 and 48 h post-transfection. RNA fractions were analyzed with primers to detect US HIV-1 RNA.

The ability of PTB to upregulate virus production without altering levels of virus gene expression or viral mRNA stability is consistent with the hypothesis that PTB acts post-transcriptionally in a direct or indirect manner to promote the export of MS HIV-1 mRNAs. This in turn could permit synthesis of Tat and Rev proteins, Tat-mediated upregulation of transcription, and Rev-mediated export of the incompletely spliced HIV-1 mRNAs that encode the structural proteins needed for virion production. To confirm this notion, PTB- and mock-transfected cells were fractionated and assessed for nuclear and cytoplasmic localization of HIV-1 RNAs. Mock-transfected controls showed exclusive nuclear localization of HIV-1 RNAs, while cells expressing PTB accumulated HIV-1 RNAs in the cytoplasm within 48 h following transfection (Figure 8). Cytoplasmic relocalization of HIV-1 RNA occurred more quickly in transfected cells than in cells stimulated with PHA (Figure 1B), reflecting the rapid and robust expression of PTB upon transfection, compared with slower upregulation following mitogen stimulation. Taken together, these results indicate that PTB acts post-transcriptionally in the nucleus to facilitate export of HIV-1 transcripts to the cytoplasm prior to virus production.

### PTB Expression Is Sufficient to Reverse HIV-1 Latency and Induce Production of Replication-Competent Virus

**Figure 9:**
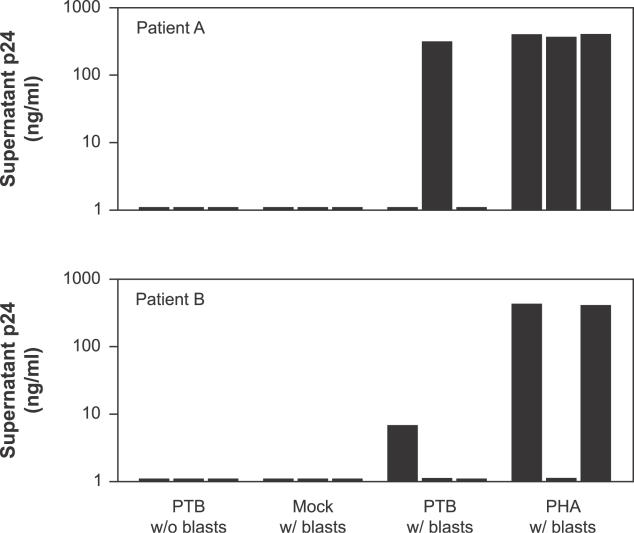
PTB Expression Is Sufficient to Reverse HIV-1 Latency and to Induce the Production of Replication-Competent Virus Resting CD4^+^ T cells from patients on HAART were transfected with empty vector, or a PTB expression vector, or were stimulated with PHA. Where indicated, cells were then co-cultured with uninfected, activated CD4^+^ T cells. Supernatant p24 levels were measured daily over a 2-wk period. Peak p24 levels are shown for each condition. Results from two representative patients are shown.

The studies described above show that the low levels of MS HIV-1 RNA found in resting CD4^+^ T cells from patients on HAART are predominantly localized to the nucleus, and that overexpression of PTB can facilitate export of these forms and induce virion production. While it cannot be definitively shown that the small subset of resting CD4^+^ T cells harboring replication-competent HIV-1 have similar patterns of RNA localization and respond similarly to PTB, the above results raise the possibility that overexpression of PTB might reverse HIV-1 latency. Therefore, we analyzed the ability of PTB overexpression to stimulate the production of replication-competent HIV-1. The release of replication-competent virus by PTB-transfected cells was measured directly in co-culture experiments using CD4^+^ T lymphoblasts from normal donors to amplify virus released from infected cells. Virus outgrowth was measured by supernatant levels of HIV-1 p24 antigen. For cells transfected with PTB, and cultured without the addition of blasts, and for mock-transfected cells co-cultured with lymphoblasts, p24 levels remained below the limit of detection in all patients studied (Figure 9). In contrast, when PTB-transfected cells were co-cultured with lymphoblasts, some wells showed readily detectable levels of supernatant p24. Results with PTB-transfected cells were similar to those seen when resting CD4^+^ T cells were stimulated with the mitogen PHA and then co-cultured with blasts, the standard method for inducing latent HIV-1 [6]. Thus, PTB expression allows the release of replication-competent virus from latently infected cells.

## Discussion

The molecular mechanism by which HIV-1 establishes a latent infection in resting CD4^+^ T cells is incompletely understood. There are profound physiologic differences between resting and activated CD4^+^ T cells [10,71–73], and it is possible that the absence of virus production in latently infected resting CD4^+^ T cells is a natural consequence of some of these differences. In fact, HIV-1 latency may not have a single mechanism but rather may result from the combined effects of incomplete blocks at multiple steps in the virus life cycle. This study defines a previously unrecognized block in virus gene expression that is unique to resting CD4^+^ T cells and that may be relevant to latency. Specifically, we show that MS HIV-1 RNAs are retained in the nucleus of resting CD4^+^ T cells. This block is particularly important because it is positioned so as to interrupt a critical positive feedback loop driven by HIV-1 Tat. We also show that overexpression of nuclear forms of the RNA-binding protein PTB can overcome this block by directly or indirectly promoting the nuclear export of HIV-1 RNAs in a manner that does not induce T-cell activation.

It is clear that HIV-1 latency is due at least in part to mechanisms that affect transcriptional initiation and elongation [21,29–41,43–49]. Resting CD4^+^ T cells from infected individuals on HAART have much lower levels of full-length HIV-1 RNA than do activated cells [49]. Interestingly, as shown here and elsewhere [21], overexpression of Tat from a non-HIV vector is sufficient to induce virus production from quiescent CD4^+^ T cells. Since Tat acts downstream of transcriptional initiation, the failure of resting CD4^+^ T cells from patients on HAART to produce virus may not be the result of a general inaccessibility of integrated proviruses to the transcriptional machinery. This conclusion is consistent with recent studies of sites of HIV-1 integration [24,32,74,75], which are generally located within introns of active cellular genes. Taken together, these results indicate that some initiation of HIV-1 transcription does occur in infected resting CD4^+^ T cells, and thus additional blocks operating downstream of transcriptional initiation must contribute to the absence of virion production.

One of the most important factors in the failure of infected resting CD4^+^ T cells to produce virions is likely to be the premature transcriptional termination resulting from the absence of Tat and associated cellular factors required for processive transcription from the HIV-1 LTR [43–47]. Short prematurely terminated transcripts can be demonstrated in resting CD4^+^ T cells from infected individuals [21,48,49]. However, although the majority of HIV-1 transcripts made in resting cells terminate prematurely, improved methods have allowed the detection of processive transcripts, including MS HIV-1 RNAs, at extremely low levels in resting CD4^+^ T cells from patients on HAART [49]. Since these mRNAs should give rise to the positive regulatory factors Tat and Rev, it is important to understand why no positive feedback upregulation of HIV-1 gene expression occurs.

To address this question, we examined the subcellular localization of MS and US HIV-1 RNAs in resting CD4^+^ T cells isolated from patients on HAART. Both species of HIV-1 RNA were localized to the nucleus of these cells. The finding that MS HIV-1 mRNAs remain in the nucleus of resting CD4^+^ T cells has not been previously reported. This result is important because it helps to explain why positive feedback does not occur and defines an additional block at the post-transcriptional level, which prevents expression of all viral protein products. This post-transcriptional block does not appear to be a global defect in resting CD4^+^ T cells and is specific to HIV-1 RNAs (Figure 1A).

To understand the nature of this block, a proteomic screen for proteins binding MS HIV-1 RNA was performed using a robust approach that successfully identified many proteins previously known to bind HIV-1 RNA or to be general RNA-binding proteins. PTB was studied as a potential positive modulator of HIV-1 gene expression due to its ability to bind MS HIV-1 RNA as well as its differential regulation in resting and activated CD4^+^ T cells. When overexpressed in resting CD4^+^ T cells from patients on HAART, PTB was sufficient to allow for high-level virus production without inducing cellular stimulation. This effect was specific to PTB, as other known HIV-1 RNA-binding proteins tested had no effect on virus production. The effect was also specific to nuclear forms of PTB as overexpression of PTBT, a cytoplasmic isoform, had no effect on virus production.

The delineation of this export block required the analysis of HIV-1 gene expression in primary CD4^+^ T cells. Although many elegant studies of latency have been carried out in transformed cell lines, these cells do not fully recapitulate the profoundly quiescent G_0_ state of the resting CD4^+^ T cells that harbor latent HIV-1 in vivo. This block was not detected previously because few studies have examined the localization of HIV-1 RNAs in primary resting CD4^+^ T cells, cells in which the levels of nuclear forms of PTB are low. Interestingly, the block can be overcome by overexpression of Tat from a non-HIV vector. In this situation, Tat-mediated upregulation of transcriptional elongation allows virus production. Thus, it may be that the nuclear export block observed in resting cells is saturable, or that it can be overcome when levels of HIV-1 transcripts increase beyond a threshold value. Partial knockdown of PTB in activated CD4^+^ T cells did not affect virus production. Thus, the clinical significance of this finding is likely to be in novel approaches for inducing virus gene expression in resting CD4^+^ T-infected cells (purging the latent reservoir) rather than in inhibiting virus production by fully activated cells.

Delineation of the mechanism for the nuclear retention of MS HIV-1 RNA in resting CD4^+^ T cells, and of the PTB reversal of this block, remains to be accomplished and may require the development of a more tractable system where levels of HIV-1 RNA can be more easily modulated. The nuclear retention of MS HIV-1 RNAs in resting CD4^+^ T cells may result from inefficient transcription by RNA pol II complexes that are not properly phosphorylated on the C-terminal domain due to the absence of Tat and associated host factors. This inefficient transcription could result in improper processing of the nascent HIV-1 mRNAs. Although HIV-1 mRNAs in resting cells appear functional by sequence analysis, assembly of downstream cellular RNA export factors may be disrupted, resulting in nuclear retention of these RNAs. PTB binds MS HIV-1 RNA and acts post-transcriptionally to overcome this obstacle and therefore allows for virus production. Whether the PTB-mediated reversal of HIV-1 latency is a direct consequence of PTB binding to HIV-1 RNA remains to be determined. Indeed, it is not possible to assert from the results presented here that PTB reverses HIV-1 latency by allowing the nuclear export of MS HIV-1 RNA. It is clear that PTB overexpression can alter the distribution of the majority of HIV-1 RNAs in resting CD4^+^ T cell populations from patients on HAART. It is also clear that PTB overexpression can induce latently infected cells in these populations to release replication-competent virus. However, it remains formally possible that these are two separate effects. In other words, PTB may be altering the distribution of HIV-1 RNAs in the majority of infected resting CD4^+^ T cells but reversing latency through a separate, as yet unidentified mechanism. Despite this caveat, it remains clear that PTB can induce the release of replication-competent virus from resting CD4^+^ T cells.

Efforts to eliminate a latent reservoir have focused on strategies that induce some level of T-cell activation in order to render cells permissive for HIV-1 gene expression [16–21,23]. Because of the potential toxicities associated with non-specific T-cell activation, there has been interest in approaches that would selectively induce viral gene expression [20,21]. Thus, obtaining a greater understanding of the mechanism of this PTB effect may contribute to the development of strategies for targeting this critical viral reservoir without inducing global T-cell activation.

## Materials and Methods

### Patients.

Resting CD4^+^ T cells were obtained from asymptomatic, HIV-1-infected adults who had suppression of viremia to below 50 copies of HIV-1 RNA/ml on HAART for at least 6 mo.

### RNA isolation and RT-PCR.

Total RNA was isolated from 10^6^ purified resting CD4^+^ T cells (described below) using the RNeasy Mini Kit (Qiagen, Valencia, California, United States). To obtain nuclear and cytoplasmic extracts, cells were lysed on ice for 5–10 min in lysis buffer containing 10 mM TrisCl [pH 7.5], 140 mM NaCl, 5 mM KCl, 1% NP-40, 1,000 U/ml RNase inhibitor, and 1 mM DTT. Lysates were centrifuged at 270 *g* for 5 min at 4° to pellet nuclei. Cytoplasmic supernatants were removed and centrifuged at 13,000 *g* for 5 min to clear any contaminating nuclei. Nuclei were incubated in RNeasy lysis buffer for 5 min on ice and centrifuged at 270 *g* for 5 min. RNA was then isolated from cytoplasmic and nuclear fractions using RNeasy. All RNAs were DNase-treated with DNase I (Amplification Grade, Invitrogen, Carlsbad, California, United States). RT-PCR was performed as described previously using hemi-nested PCR primer sets US, E2, and E5 [49]. All RT-PCR experiments included control reactions set up without the addition of RT, as well as no template controls. All such controls were negative. Real-time RT-PCR was performed using the Platinum Quantitative RT-PCR ThermoScript One-Step System (Invitrogen) according to the manufacturer's protocol. Primers and probe for quantitative RT-PCR were as follows: QPCR LTR3′: 5′ TAAAAGGGTCTGA-GGGATCTCTAGTT3′, QPCR LTR5′: 5′ GCCTCAATAAAGCTTGCCTTGA3′, probe: 5′ FAM-AGTCACACAACAGACGGGCACACACTACTT-BlackHole Quencher1–3′. Probes were obtained from Biosource International. The quantitative real time RT-PCR has a limit of detection of 10–100 copies. The semi-quantitative method has a limit of detection of ~10 copies.

### In vitro HIV-1 RNA-binding assays.

MS HIV-1 RNA was cloned from RNA isolated from patient PBMCs (peripheral blood mononuclear cells). Reverse transcribed HIV-1 RNA containing exons 1, 5, and 7 was cloned into pSP64 Poly(A) (Promega, Madison, Wisconsin, United States). MS HIV-1 RNA was produced by in vitro transcription using the RiboMAX™ Large Scale RNA Production System (Promega) according to manufacturer's protocol. The resulting RNA was incubated with an equimolar amount of a biotinylated oligo-dT probe coupled to streptavidin magnetic particles (Roche, Basel, Switzerland). The RNA-coated beads were incubated with lyastes (50 μg) of purified resting or activated CD4^+^ T cells. RNA-binding proteins were identified by PAGE MS/MS using the mass spectrometry facility of Dr. Bill Lane at Harvard Medical School. For recombinant protein binding assays, 50 ng of protein was used.

### Recombinant proteins.

cDNA encoding full-length PTB isoform 1 (kind gift of S. Bouyain and B. Geisbrecht) was cloned into the pT7 vector, which encodes an N-terminal poly-histidine tag followed by a Tobacco Etch Virus cleavage site. Recombinant protein was expressed in Rosetta pLysS expression hosts (EMD Biosciences), and soluble protein was purified using nickel-loaded Chelating Fast Flow resin (Amersham Biosciences, Little Chalfont, United Kingdom). The partially purified protein was cleaved using recombinant TEV protease (Invitrogen), and fully cleaved protein from a reverse IMAC purification was further purified to near homogeneity using a Superdex S-200 gel filtration column (Amersham Biosciences). Protein concentration was determined using the UV spectroscopy at 278 nm.

### Antibodies, Western blots, and immunofluorescence.

Anti-PTB was obtained from Zymed (San Francisco, California, United States). For Western blots, a 1:350 dilution of antibody was used, and for immunofluorescence, a 1:250 dilution was used. Both La and hnRNPA1 antibodies were used at a concentration of 0.5 μg/ml for Western blots. 20 μg of total protein were loaded per lane for Western blots. For immunofluorescence, AlexoFluor596 (Molecular Probes, Eugene, Oregon, United States) was used as a secondary reagent according to manufacturer's protocol. OregonGreen 488 phalloidin was used for actin staining according to manufacturer's protocol (Molecular Probes). Nuclear staining was performed with DAPI (Molecular Probes) using ProLong Gold antifade reagent. Images were taken using a Perkin Elmer (Wellesley, Massachusetts, United States) UltraVIEW Spinning Disk Confocal Microscope.

### T-cell purification and transfections.

Resting CD4^+^ T cells were isolated through a two-step purification procedure as previously described [6,49]. To obtain activated CD4^+^ T cells, Ficoll-purified PBMCs were stimulated with PHA for 3 d, and CD4^+^ T cells were positively selected using the Dynal (Oslo, Norway) CD4^+^ selection kit. Transfections were performed using an Amaxa Nucleofector. 2–4 × 10^6^ purified CD4^+^ T cells were re-suspended in 100 μl/10^6^ cell in nucleofector solution and transfected using program U-14. The number of cells used depended on the number of cells obtained after purification. Cells were cultured in 1.5 ml medium after transfection. For a given patient, the number of cells used was identical for each condition. For each transfection, 2.5 μg of empty vector or plasmid with PTB, PTBS-A, PTBT, or pcDNA-TAT-86 was transfected with 2.5 μg of plasmid phMGFP (Promega). GFP expression served as a control for transfection efficiency. For RNA-turnover experiments, α-amanitin was added to medium at a final concentration of 50 μg/ml 24 h after transfection. Total RNA was isolated at various time points after α-amanitin addition. PTB and PTBT were cloned from PBMC cDNA using the following primers: 5′ACGCGTCGACAATGGACGGCATTGTCCCAGATATAGCC3′ and 5′ATAGAATGCGGCCGCCTAGATGGTGGACTTGGAGAAGG3′. PCR products were cloned using SalI and NotI restriction sites into the pCI-neo mammalian expression vector (Promega). PTB ser-ala was made by direct mutagenesis of serine16 to alanine in the PTB expression vector. pcDNA-TAT-86 was a gift from Dr. Avi Nath (Johns Hopkins University, Baltimore, Maryland, United States).

### T-cell co-culture.

To isolate replication competent viruses from latently infected cells after PTB transfection, transfected cells were co-cultured with CD4^+^ lymphoblasts from uninfected donors as described previously [6]. Resting CD4^+^ T cells were isolated from patients on HAART and transfected as described above. At 24 h after transfection, lymphoblasts were added to each well at a ratio of 2:1 to amplify virus released from latently infected cells. In control wells, untransfected resting CD4^+^ T cells were activated with PHA and co-cultured with lymphoblasts as previously described [6]. Supernatants were sampled every day over a 2-wk period, and levels of p24 antigen were measured by ELISA.

## Supporting Information

Figure S1 Proteomic Screen for MS HIV-1 RNA-Binding Proteins (A) Schematic of in vitro-transcribed MS HIV-1 RNA used in binding assays. This RNA contains exons 1, 5, and 7. (B) Silver stain of MS HIV-1 RNA-binding proteins in CD4^+^ T cells. Bands that decreased in intensity with increasing molar equivalents of competitor RNA (non-biotinylated MS HIV-1 RNA) were analyzed (arrows).
(184 KB DOC)

Figure S2 Effect of siRNA Knockdown of PTB on Virus Production by Infected CD4^+^ T Lymphoblasts (A) Semi-quantitative RT-PCR of full-length PTB in cells transfected with a non-targeting siRNA or a siRNA directed against PTB. Semi-quantitative RT-PCR for GAPDH served as an input control. (B) Knockdown of PTB in activated CD4^+^ T cells had no effect on HIV-1 production. Resting CD4^+^ T cells from patients on HAART were activated with PHA and then transfected 48 h post-stimulation with a non-targeting siRNA or a siRNA directed against PTB. Cells were washed every 24 h and supernatants were analyzed for viral load 72 h post-transfection.
(36 KB DOC)

### Accession Numbers

The National Center for Biotechnology (NCBI) (http://www.ncbi.nlm.nih.gov) accession number for polypyrimidine tract binding protein (PTB) is NM_002819.3.

## Abbreviations

GAPDH: glyceraldehhyde-3-phosphate dehydrogenase
HAART: highly active antiretroviral therapy
LTR: long terminal repeat
MS: multiply spliced
NLS: nuclear localization signal
PHA: phytohemagglutinin
PTB: polypyrimidine tract binding protein
PTB ser-ala: PTB with a serine to alanine mutation
PTBT: a 25-kDa cytoplasmic isoform
siRNA: short interfering RNA
US: unspliced
