# Supplementary material for: Nuclear Retention of Multiply Spliced HIV-1 RNA in Resting CD4+ T Cells
Source: PLoS Pathog. 2006 Jul 7;2(7):e68. doi: 10.1371/journal.ppat.0020068 (PMC1487174; doi:10.1371/journal.ppat.0020068)
Supplement: Supplementary file 2 [file ppat.0020068.sg002.doc]

**Figure S2.**
